# A Hydrophilic Copper–Viologen Hybrid Exhibiting High Degradation Efficiency on Commercial Dye in Maritime Accidents

**DOI:** 10.3390/molecules30173525

**Published:** 2025-08-28

**Authors:** Yali Gao, Chaojian Hu, Xihe Huang, Haohong Li, Tong Lou, Xueqiang Zhuang

**Affiliations:** 1 School of Marine Engineering, Jimei University, Xiamen 361021, China; yali_gao@jmu.edu.cn (Y.G.); 202411824015@jmu.edu.cn (C.H.); loutong@jmu.edu.cn (T.L.); 2 Fujian Province Key Laboratory of Ship and Ocean Engineering, Xiamen 361021, China; 3 Fujian Institute of Innovation for Marine Equipment Detection and Remanufacturing Industrial Technology, Xiamen 361021, China; 4 College of Chemistry, Fuzhou University, Fuzhou 350116, China; xhhuang@fzu.edu.cn (X.H.); lihh@fzu.edu.cn (H.L.)

**Keywords:** visible-light-driven catalysis, copper-based photocatalyst, asymmetric viologen, photocatalytic degradation

## Abstract

Photocatalysis is a promising strategy for the treatment of dangerous chemical pollutants in the ocean. In this work, a stable copper-based photocatalyst, i.e., {[Cu(BPA)_2_]·2I_3_}_n_ (**1**, BPA = 4,4′-bipyridinium-N-pentanoic acid), exhibited excellent degradation performance in dye pollutant in seawater. According to the structural analysis, this photocatalyst consists of 1-D cationic [Cu(BPA)_2_]_n_^2n+^ infinite chain and two I^3−^ polyiodide anions. In the [Cu(BPA)_2_]_n_^2n+^ chain, the distorted CuO_4_N_2_ octahedra are bridged by asymmetric viologen ligand (BPA), which result in a 1-D ladder-shaped chain. Strong C–H···O/I hydrogen bonds contribute to the formation of a 2-D layer along *bc*-plane, in which I^3−^ anions are stacked among the cationic chains. The strong adsorption from ultraviolet to visible regions together with its high charge separation efficiency implies its usage as excellent visible-light-driven catalysis. Interestingly, good photocatalytic performance for the degradation of Rhodamine B (RhB) in seawater can be observed by using this hybrid as photocatalyst. In detail, 90.6% degradation ratio of RhB can be achieved in 150 min under visible light, which was monitored on a UV–Vis spectrum. This work could pave the way for new ocean pollutant treatments for shipping accidents.

## 1. Introduction

Synthetic dyes have been widely used in many fields, including textile printing, plastics, and paper, the annual worldwide production of which is about 70 million tons [1]. Due to increased globalization, most synthetic dyes are conveyed by sea transportation. In the past twenty years, the number of shipping accidents has increased greatly. Therefore, the sea safety uncertainty resulting from dye seepage in sea transportation has become a severe environmental problem, which calls for a new largescale chemical pollutant treatment process [2]. In organic dyes, about 80% are azo-type dyes possessing chromophores, for example, Rhodamine B (RhB) and methyl orange (MO) [3]. Azo-type dyes are inert to biodegradation, which results in environmental problems due to biological toxicity. For example, Rhodamine B can trigger ovarian toxicity through oxidative stress, resulting in lower 17B-estradiol level and thickness of endometrium [4]. As a green technology, semiconductor-based photocatalysis has captured significant attention in the field of dye pollutant degradation, because it can completely decompose the dyes to non-toxic chemicals with high efficiency and low cost [5]. Photocatalytic semiconductors are key to the degradation efficiency. So far, several types of photocatalysts have been developed, including traditional metal oxides (TiO_2_, ZnO) [6], metal sulfides (CdS, ZnS, Mo/WS_2_) [7], bismuth oxyhalides (BiOX, X = F, Cl, Br, I) [8,9], non-metallic materials (graphene and g-C_3_N_4_) [10,11], and inorganic/organic hybrids [12,13]. For inorganic metal oxides/sulfides, the following problems have puzzled researchers for a long time: low visible light utilization degree due to the wide band gaps of catalysts, high photogenerated carrier recombination ratio, and the photo-induced instabilities [14]. Organic photo-catalysts (g-C_3_N_4_) also face problems of low active sites and photo-corrosion [15]. But inorganic/organic hybrids have attracted a special interest because these problems can be overcome by rational structural modifications [16]. For example, some Pb/Bi-based inorganic/organic hybrids with smaller band gaps have been adopted as catalysts in dye degradations with high light harvesting efficiencies [12,13,17]. However, they are environmentally unfriendly; thus, searching for environmentally friendly crystalline inorganic/organic photocatalysts with tailorable structures is desirable. Copper–viologen hybrid might solve the above problems because the high electron-accepting ability of viologens and strong coordinated Cu-involved coordinated bonds [14]. Furthermore, significant effort has been made regarding the degradation of azo-type dyes in fresh water, but the treatments for seawater are still rare and many scientific problems remain unclear [18,19,20,21]. For example, the effect of halogen ion (Cl^−^ ion) and metal ions (Na^+^, Mg^2+^, Ca^2+^ et al.) on seawater during the degradation processes are in their infancy. It was found that the photooxidative decolorization of RhB was accelerated in the presence of a certain amount of salt [22], and the presence of metal cations can enhance the photocatalytic activity [20]. In this work, a stable copper-based photocatalyst, i.e., {[Cu(BPA)_2_]·2I_3_}*_n_*(**1**, BPA= 4,4′-bipyridinium-N-pentanoic acid), exhibits excellent degradation performance on dye pollutants in seawater, and the mechanism of this is also discussed.

## 2. Results and Discussion

### 2.1. Structural Characterization of Photocatalyst

**Figure 1:**
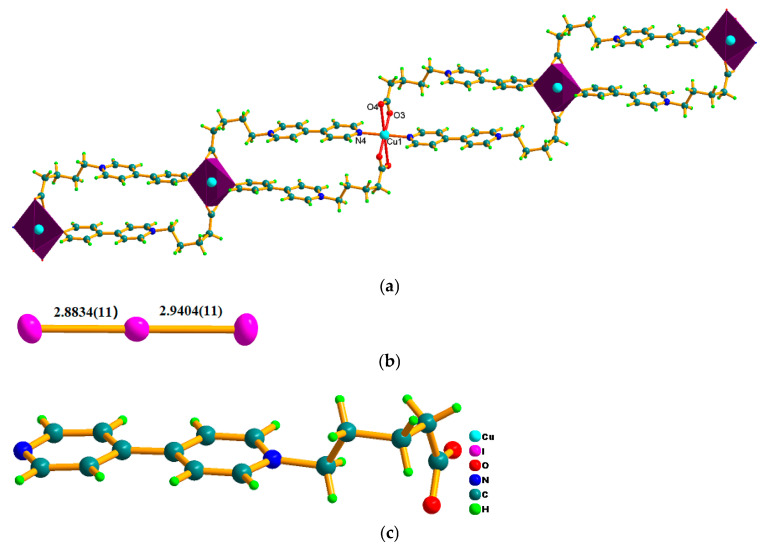
(**a**) A 1-D ladder-shaped cationic [Cu(BPA)_2_]_n_^2n+^chain; (**b**) the symmetrical molecular triiodide in the I_3_^−^ ion; (**c**) the structure of BPA ligand.

**Figure 2:**
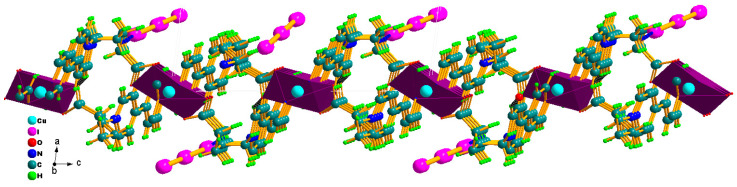
The 2-D layer along *bc*-plane base on C–H···O () and C–H···I hydrogen bonds.

This photocatalyst consists of the 1-D cationic [Cu(BPA)_2_]_n_^2n+^ infinite chain and two isolated I_3_^−^ polyiodide anions, strong C–H···I hydrogen bonds contribute to the stable hybrid structure. In the [Cu(BPA)_2_]_n_^2n+^ cationic chain, copper centers are generally found in ideal CuO_4_N_2_ octahedral geometry, in which four O donors stemming from two carboxyl groups are on the equator plane and two N donors from two pyridine rings occupy the axial positions (Figure 1a). The Cu–O/N distances of 1.951(7)~2.035(7) Å imply strong coordinated bonds, and the octahedral angles of 88.3(3)~91.3(3) and 180.000(1)° are consistent with the ideal octahedron (Table S1). Neighboring CuO_4_N_2_ octahedra are bridged by the asymmetric viologen ligand (BPA) to yield a 1-D ladder-shaped chain (Figure 1a). The size of ladder defined by two BPA ligands and CuO_4_N_2_ octahedra is about 4.71 × 12.88 Å. Two I_3_^−^ ions are molecular triiodide with symmetrical I–I distances and linear I–I–I angles. For example, bond lengths/angle in I(1)–I(2)–I(3) are 2.8835(10)/2.9404(10) Å and 179.46(3)° [13]. In the BPA ligand, the carboxylate group presents a bis-dentate chelated mode with averaged C-O bonds (1.234(11), 1.290(11) Å) (Figure 1c). The dihedral angles between two pyridine rings in BPA cations is 3.819°, suggesting a co-planar nature. The C–C–C angles of the alkyl chain are 110.24~116.68°, deviating clearly from 109.28° for sp^3^ hybrid C atom. The larger C–C–C angles are driven by the coordination of carboxyl group. Strong C–H···O hydrogen bonds among [Cu(BPA)_2_]_n_^2n+^ chains with carboxylic O as acceptors contribute to the formation of a 2-D layer along the *bc*-plane, and I_3_^−^ ions are anchored in the layer via C–H···I hydrogen bonds (Table S2, Figure 2). The strong coordinated bonds and C–H···I hydrogen bonds together with good co-planar nature of viologen ligands are beneficial for its excellent stability and high charge separation efficiency as a photocatalyst. The coordinated polymers constructed from asymmetric viologen are still rare [12]. In particular, the 1-D ladder-like chain constructed from carboxyl-bearing asymmetric viologen is unique due to its combination of rigidity and flexibility, which is solution-stable [23].

### 2.2. Visible Absorption Behavior and Transient Photocurrent Response

**Figure 3:**
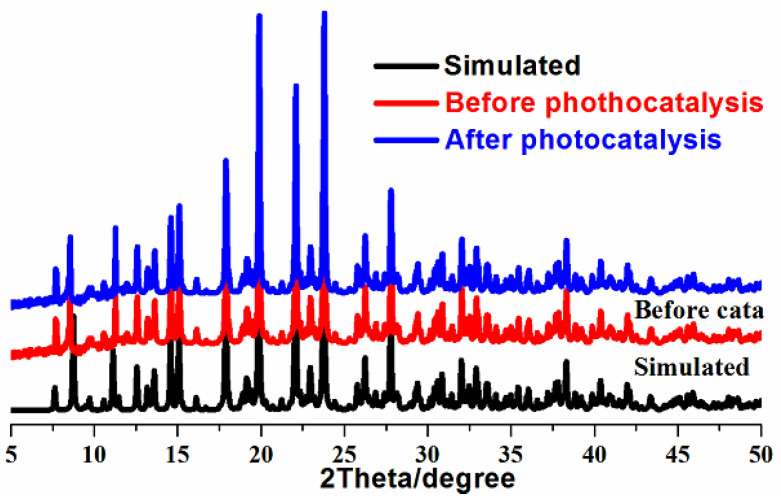
Powder X-ray diffraction (PXRD) patterns of catalyst 1 (black: simulated; red: as-synthesized catalyst before photocatalysis; blue: catalyst after photocatalysis).

**Figure 4:**
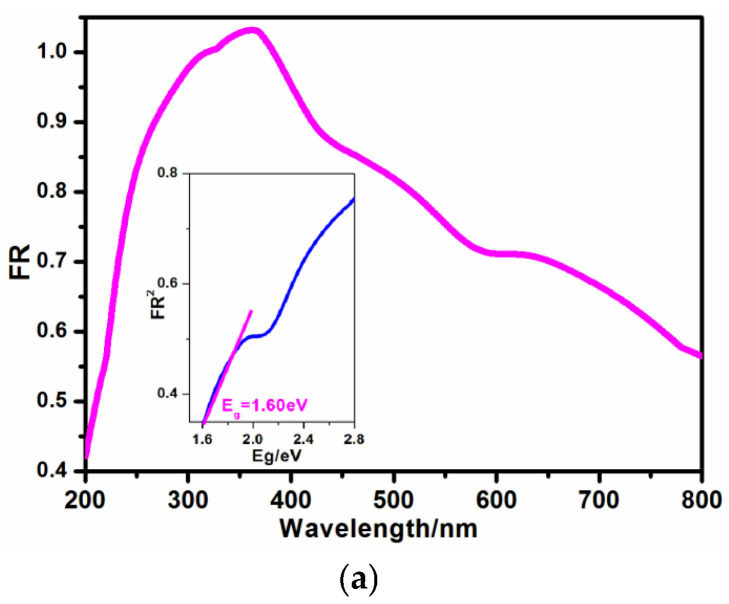

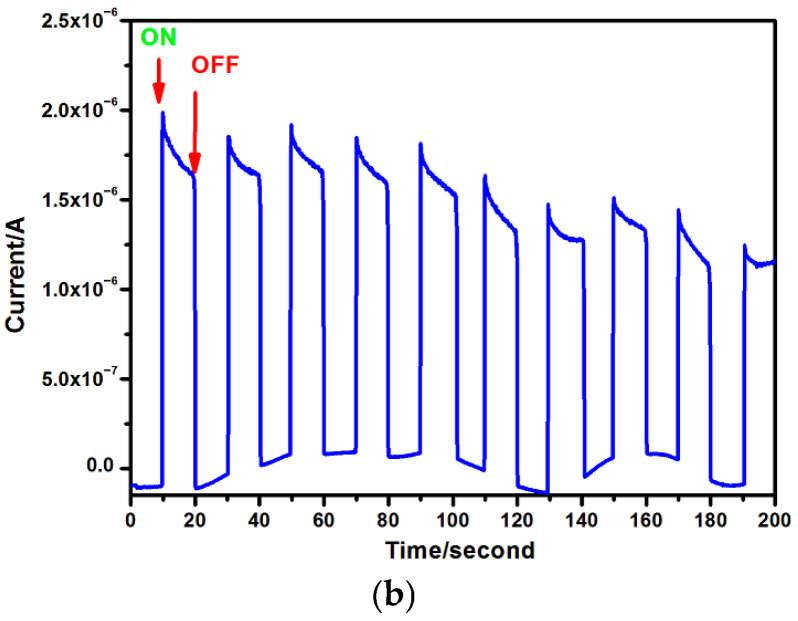
(**a**) Absorbance spectrum of photocatalyst (insert: K-M fitting showing its optical gap, orange: absorbance spectrum; blue: optical gap); (**b**) transient photocurrent response of photocatalyst.

Powder X-ray diffraction (PXRD) was conducted to verify the purity of crystalline catalyst **1**. As shown in Figure 3, the diffraction patterns of newly as-synthesized catalyst are consistent with those of simulated ones based on single crystal X-ray diffraction, hinting at its good phase purity. A UV–Vis spectrum of **1** was executed to obtain its adsorption domains, as illustrated in Figure 4a. Interestingly, a wide absorption zone ranging from ultraviolet to visible regions (250~800 nm) can be observed in photocatalyst **1**, demonstrating its application capabilities as a visible light-driven photocatalyst. The absorption zone is much wider than those of the reported viologen-based hybrids [24]. The strong adsorption at 320 and 354 nm stem from the n/π-π* charge transfer of BPA ligand [13,21], and the relatively weak adsorption at 614 nm could be a result of the intermolecular charge transfer between the I_3_^−^ ions and the BPA ligand, i.e., the I-5p to the π* orbital of BPA cations [25]. In all, the visible and near-infrared absorptions of **1** could be relative to charge or partial electron transfers among [Cu(BPA)_2_]_n_^2n+^ chains and I_3_^−^ ions, which are assisted by strong hydrogen bonds. Therefore, the photocatalytic degradation of organic dye using this photocatalyst was driven by visible light. The optical gap (*E*_g_) calculated using the Kubelka–Munk function has been widely adopted in the characterization of the photocatalyst, which is a simple and reliable method [26]. The optical gap (*E*_g_) of **1** was estimated to be 1.60 eV, which is relatively narrow (Figure 4a, inserted) [27].

The charge separation efficiency of this photocatalyst was estimated by transient photocurrent response measure (Figure 4b), during which the photocatalyst-coated ITO electrode was used as working electrode. The light resource was a 150 W xenon lamp, and the electrode was irradiated using an ON–OFF cycle with intervals of 10 s. It is clear that repeatable photocurrents with rapid responses are present, whose photocurrent density is about 1.62 μA·cm^−2^. The light current drop with elongated irradiating time might be relative to the asymmetric I_3_^−^ anions in the lattice [12]. The small baseline fluctuation might be a result of un-stable contact resistance on working electrode. This photocurrent data confirms the low recombination rate and the highly efficient separation of photogenerated electron–hole pairs in this photocatalyst. The wide absorption band in the visible zone or narrow optical gap ultimately result in high levels of photoactivity [28].

### 2.3. Photocatalytic Degradation of RhB in Seawater

**Figure 5:**
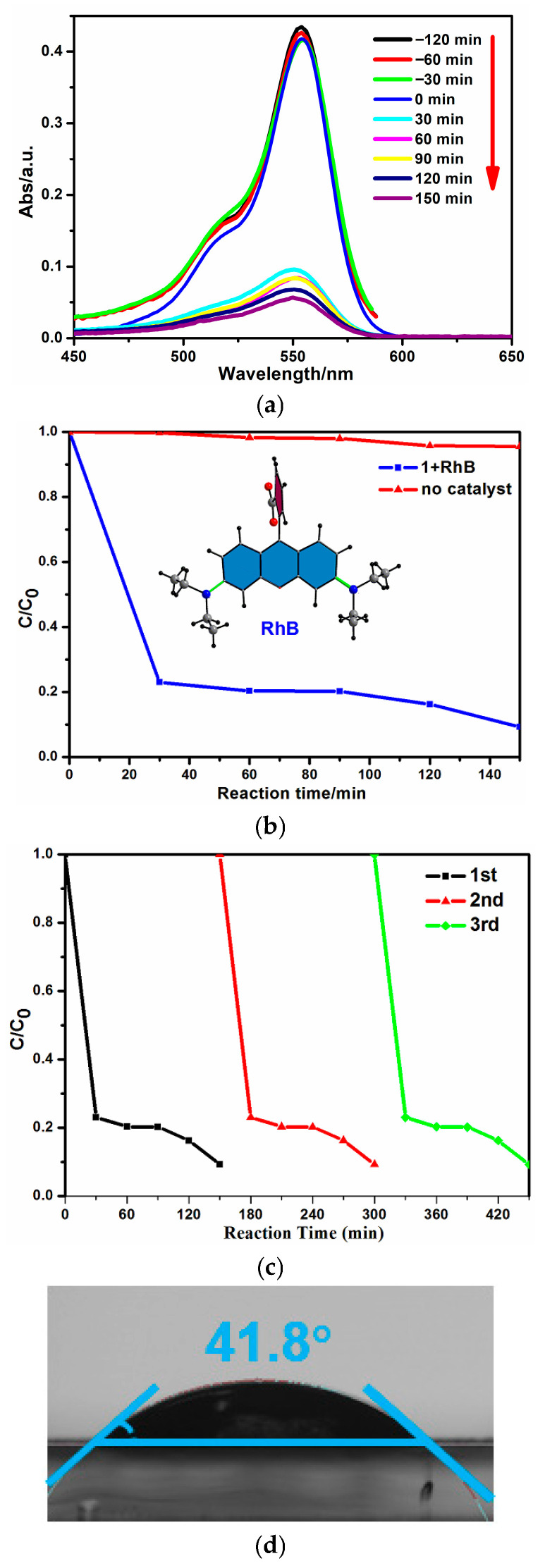
(**a**) UV–Vis spectra of RhB solution with the presence of photocatalysts at different degradation times; (**b**) degradation ratio of RhB by elongating the irradiation time with or without the presence of a catalyst; (**c**) recycling tests of 1 for RhB photodegradation under light irradiation; (**d**) water contact angle.

The dye pollutants in seawater (using RhB as model) can be efficiently degraded by the photocatalytic method with this copper-based catalyst, and the RhB photolysis without the photocatalyst is given as reference (Figure 5b). With the absence of photocatalyst, the photolysis of RhB using only visible light irradiation is generally negligible. But with the presence of photocatalyst **1**, the concentrations of RhB in seawater decreased quickly under visible light irradiation, which were monitored using time-dependent UV–Vis spectra (Figure 5a). At an irradiation time of 150 min, the absorption intensities of RhB were reduced greatly, hinting at the degradation of RhB. Degradation efficiency can reach 90.6% at an irradiation time of 150 min, which could be comparable to {[(BiI_6_)I_13_]·2I_3_·(H-BPA)_4_}_n_ [21], but better is than that of (PBPY-H_2_)_2_[PbI_4_(I_3_)_2_] [13]. The rate constant calculated from the UV–Vis was 0.0134 min^−1^. This stable copper-based catalyst can be reused, which was proven by recovering solids from solution using a centrifugal method (the degradation reaction is heterogeneous). The recovered catalyst was treated by washing (ethanol) and vacuum-drying at 60 °C. The recovered ratio was as high as 94.6% at the recovered amount of 37.84 mg, and the small amount of loss could be a result of its unavoidable solubility in seawater. Then, PXRD measurements were conducted on this recovered catalyst, its XRD patterns are nearly identical with those of fresh sample (Figure 3), suggesting that this catalyst could be stable in seawater. Furthermore, the photocatalytic activity can be maintained after three degradation cycles (Figure 5c). More degradation cycles might lead to its deactivation, which should be relative to the instability of catalyst in seawater, during which Cu–O coordinated bonds could be destroyed. The strategy for improving the stability of this type of catalyst could enhance its hydrophobicity by introducing some hydrophobic groups. This hybrid exhibits good hydrophily, judging by its water contact measurement (41.8°, Figure 5d), thus implying better catalyst–water interactions during seawater pollutant degradation.

The photocatalytic mechanism of this viologen-containing system could be proposed as follows: the electrons of the photocatalyst can transfer from the valence bands (VBs) to the conduction bands (CBs) under visible light irradiation. Consequently, the hydroxide free radical ˙OH can be generated during the capturing of the holes (h^+^) in the VBs by OH^−^ in the system, and the superoxide radical anion ˙O^2−^ could be formed simultaneously through the trapping of excited electrons by O_2_ [29,30]. Due to the presence of the strong electronic acceptor nature of viologen, ˙O^2−^ may be the dominant species in the photodegradation process [12,16]. In all, this copper-based compound can be used as highly efficient photocatalyst for the decomposition of RhB in seawater under visible light irradiation. It is universally accepted that in the photocatalytic degradation of RhB, N-de-ethylation, chromophore cleavage and mineralization can occur, and the final products are CO_2_, H_2_O, NO^3−^, and NH^4+^ [31]. According to the TOC analysis on degradation solution, a removal ratio of 91.2% hints at the degradation of RhB.

## 3. Experiment

### 3.1. Materials and Methods

All the chemicals except 4,4′-bipyridinium-N-pentanoic acid (BPA·Br) were reagent-grade and were used as purchased. BPA·Br was self-prepared using 4,4′-bipyridine and 5-bromo-pentanoic acid as starting materials and CH_3_CN as a solvent, during which the reaction was maintained at 40 °C for 24 h [27]. IR spectra were recorded on a Perkin-Elmer Spectrum-2000 FTIR spectrophotometer (4000~400 cm^−1^). The solid-state diffuse reflectance spectrum was measured on a UV–Visible spectrometer (Thermo SCIENTIFIC, Waltham, MA, USA, EVLUTION 220). Elemental analysis for C, H, and N was performed on a Vario MICRO elemental analyzer. The optical gap was calculated from the diffuse reflectance data using the Kubelka–Munk function: α/S = (1 − R)^2^/(2R), in which α was the absorption coefficient, S was scattering coefficient, and R was reflectance [32]. An elemental analysis for C, H, and N was carried out on a Vario MICRO elemental analyzer. The crystalline purity of the catalyst was characterized by powder X-ray diffraction (XRD, D8-advanced, Bruker, Karlsruhe, Germany, CuKα radiation). The photocurrent data were obtained using a CHI650 electrochemistry workstation with a three-electrode system. Total organic carbon (TOC) was determined using Shimadzu TOC-L_CPH-CPN_ equipment.

### 3.2. Synthesis of Photocatalyst {[Cu(BPA)_2_]·2I_3_}_n_ (1)

BPA·Br (0.1120 g, 0.33 mmol), CuI (0.0500 g, 0.33 mmol), and I_2_ (0.0600 g, 0.33 mmol) were dissolved in 5 mL H_2_O. The suspension was then transferred into a 25 mL Teflon-lined autoclave and was stirred for 90 min until a clear solution was obtained. Afterwards the autoclave was sealed and heated to 120 °C for 100 min, and the resultant mixture was held at this temperature for 72 h. Finally, the autoclave was cooled to room temperature (30 °C) in 24 h. Red block single crystals with sizes of about 0.2~0.4 mm were isolated and washed with ether three times (0.1015 g, yield 23% based on Cu). Anal. Calcd. for C_30_H_32_CuI_6_N_4_O_4_ (1337.54): C, 26.94; H, 2.39; N, 4.19%. Found: C, 27.15; H, 2.42; N, 4.25%. IR (KBr, cm^−1^): 3425 (w), 3105 (w), 2926 (w), 1630 (w), 1605 (m), 1580 (s), 1563 (s), 1454 (w), 1417 (s), 1400 (s), 1330 (w), 1216 (s), 1172 (m), 1073 (m), 610 (s), 812 (s), 713 (m), 494 (m).

### 3.3. X-Ray Crystallography

The intensity data were collected on a Bruker APEX II diffractometer using graphite-monochromated Mo*Kα* radiation (*λ* = 0.71073 Å) at room temperature. Within the range of 1.28 ≤ *θ* ≤ 25.02°, of the 11,462 total reflections, 6490 were independent with *R*_int_ = 0.0357, of which 5382 were observed (*I* > 2σ(*I*)) and used in the succeeding refinement. The correction of multi-scan absorption corrections was applied. The structure was solved using the direct method and expanded through the Fourier technique using the SHELXTL-97 program package [33,34]. Non-hydrogen atoms were refined with anisotropic thermal parameters, and hydrogen atoms were assigned with common isotropic displacement factors and included in the final refinement by using geometrical constraints. The simulated XRD patterns were obtained by using its cif file based on Mercury program (3.10.2 version). Crystal data: Triclinic, space group *P*-1 with *M_r_* = 1337.54, *a* = 10.3594(15), *b* = 11.7585(16), *c* = 16.054(2) Å, *α = * 89.582(3), *β* = 82.243(2), *γ* = 80.146(2)°, *V* = 1908.8(4) Å^3^, *Z* = 2, *D_c_* = 2.327 g/cm^3^, *F*(000) = 1238, *μ*(Mo*Kα*) = 5.462 mm^−1^, the final *R* = 0.0610, *wR* = 0.1736, *S* = 1.122, (Δ/*σ*)_max_ = 0.000, (Δ*ρ*)_max_ = 1.517, and (Δ*ρ*)_min_ = −2.144 Å^3^. Selected bond lengths and bond angles are given in Table S1, and C–H···π interactions are shown in Table S2. Crystallographic data for **1** were deposited at the Cambridge Crystallographic Data Center (CCDC 1448835).

### 3.4. Photocurrent Measurements

Photocurrent measurements were conducted to determine the photo-responsive and charge separation performance of the as-synthesized photocatalyst, in which the solution method was used in the fabrication of electrodes [35]. The newly prepared crystalline photocatalyst (5 mg) was dispersed in 0.5 mL DMF (N,N-dimethylformamide), and the resultant solution was spread on ITO glass (0.36 cm^2^), which was precleaned with acetone. Epoxy resin was used to cover the uncoated parts of ITO. The electrode can be fabricated by evaporating the solvent under an ambient atmosphere. A 150 W xenon lamp equipped with a cutoff filter (>420 nm) was used as a light resource to determine its photo-responsive behavior. Photocurrent data were plotted by an electrochemical workstation in a typical three-electrode system in a NaSO_4_ aqueous solution (0.2 M): the **1**-coated electrode as a working electrode, the platinum plate as counter electrode, and a Ag/AgCl electrode as reference electrode. The applied voltage was set as 0.5 V, which was determined by the narrow gap of 1.60 eV. The lamp was turned on throughout the measurement, and the irradiation was blocked by a manual shutter at intervals of 10 s.

### 3.5. Photodegradation of RhB

Artificial seawater was prepared according to the literature (3.4 wt% containing NaCl (77.9%), MgCl_2_ (9.6%), MgSO_4_ (6.1%), CaSO_4_ (4.0%), and KCl (0.07%) [36]. In a typical RhB degradation experiment, a 300 W Xe arc lamp equipped with a λ ≥ 420 nm cutoff filter and an IR filter is set as visible light source, whose output light intensity are measured as 110 mw/cm^2^. A 40 mg photocatalyst was suspended in 80 mL RhB solutions (concentration: 10 ppm). In order to achieve the adsorption–desorption equilibrium of the organic contaminants on the photocatalyst surfaces, before irradiation, the suspensions were magnetically stirred in the dark for 2 h until the UV/Vis absorption of the RhB solution did not change (Figure 5a). The photocatalytic performance of the catalysts was estimated by monitoring the UV/Vis absorbance (at λ = 555 nm) characteristic of the target dye (RhB). A total of 3 mL of sample solutions were taken out at given time intervals and were separated through sample filtration. The residual concentrations of RhB in solution were monitored by recording the UV–Vis absorption density variations at 553 nm, which was the characteristic absorption of RhB. The percentage of degradation is reported as *C*/*C*_0_. Here, *C* is the absorption of RhB at each irradiated time interval of the main peak of the absorption spectrum at 553 nm, and *C*_0_ is the absorption of the starting concentration when adsorption–desorption equilibrium is achieved. The reference experiment without the catalyst was conducted for benchmarking.

## 4. Conclusions

In summary, a stable copper-based photocatalyst, i.e., {[Cu(BPA)_2_]·2I_3_}_n_, was synthesized and structurally determined and it consisted of a 1-D cationic [Cu(BPA)_2_]_n_^2n+^ infinite chain and two I_3_^−^ polyiodide anions. Its quasi-2-D layer was constructed from strong C–H···O/I hydrogen bonds. This photocatalyst can be used in the degradation of rhodamine B in seawater driven by visible light, which can be attributed to its wide absorption zone and high charge separation efficiency. The degradation ratio of RhB can reach 90.6% in 150 min. In addition, this catalyst exhibits good stability and reusability. This stable and cheap photocatalyst could be potentially used in chemical pollutant treatment in shipping accidents.

## Data Availability

Data are contained within the article or the Supplementary Materials.

## Supplementary Materials

The following supporting information can be downloaded at: https://www.mdpi.com/article/10.3390/molecules30173525/s1. Data S1: cif file for publication. Table S1: Selected Bond Lengths (Å) and Bond Angles (°) of catalyst, Table S2: Hydrogen Bond Lengths (Å) and Bond Angles (°).
