# Supplementary material for: A Hydrophilic Copper–Viologen Hybrid Exhibiting High Degradation Efficiency on Commercial Dye in Maritime Accidents
Source: Molecules. 2025 Aug 28;30(17):3525. doi: 10.3390/molecules30173525 (PMC12430637; doi:10.3390/molecules30173525)
Supplement: Supplementary file 1 [file molecules-30-03525-s001.zip › molecules-3749879-supplementary.pdf]

Electronic supporting information

A Hydrophilic Copper–Viologen Hybrid Exhibiting High Degradation Efficiency on Commercial Dye in Maritime Accidents

Yali Gao [1,2,3], Chaojian Hu [1,2,3], Xihe Huang [4], Haohong Li [4], Tong Lou [1,2,3] and Xueqiang Zhuang [1,2,3,*]

[1] School of Marine Engineering, Jimei University, Xiamen 361021, China
[2] Fujian Province Key Laboratory of Ship and Ocean Engineering, Xiamen 361021, China
[3] Fujian Institute of Innovation for Marine Equipment Detection and Remanufacturing Industrial Technology, Xiamen 361021, China
[4] College of Chemistry, Fuzhou University, Fuzhou 350116, China
* Correspondence: xqz@jmu.edu.cn

Table S1. Selected Bond Lengths (Å) and Bond Angles (°) of catalyst

Bond	Dist.	Bond	Dist.	Bond	Dist.
Cu(1)–O(3)	1.951(7)	Cu(1)–O(3)[a]	1.951(7)	Cu(1)–N(4)	2.035(7)
Cu(1)–N(4)[a]	2.035(7)	Cu(2)–O(1)[b]	1.958(6)	Cu(2)–O(1)	1.958(6)
Cu(2)–N(1)	2.016(7)	Cu(2)–N(1)[b]	2.016(7)	I(1)–I(2)	2.8835(10)
I(2)–I(3)	2.9404(10)	I(4)–I(5)	2.8880(10)	I(5)–I(6)	2.9347(10)
O(1)–C(30)	1.290(11)	O(2)–C(30)	1.234(11)	O(3)–C(15)	1.267(13)
O(4)–C(15)	1.234(13)				
**Angle**	**(°)**	**Angle**	**(°)**	**Angle**	**(°)**
O(3)–Cu(1)–O(3)[a]	180.000(1)	O(3)–Cu(1)–N(4)	88.7(3)	O(3)[a]–Cu(1)–N(4)	91.3(3)
O(3)–Cu(1)–N(4)[a]	91.3(3)	O(3)[a]–Cu(1)–N(4)[a]	88.7(3)	N(4)–Cu(1)–N(4)[a]	180.000(1)
O(1)[b]–Cu(2)–O(1)	180.000(1)	O(1)[b]–Cu(2)–N(1)	91.7(3)	O(1)–Cu(2)–N(1)	88.3(3)
O(1)[b]–Cu(2)–N(1)[b]	88.3(3)	O(1)–Cu(2)–N(1)[b]	91.7(3)	N(1)–Cu(2)–N(1)[b]	180.000(1)
I(1)–I(2)–I(3)	179.46(3)	I(4)–I(5)–I(6)	179.25(3)		

Symmetry transformation: a -x,-y-1,-z+2; b -x,-y+1,-z+

Table S2. Hydrogen Bond Lengths (Å) and Bond Angles (°)

D–H···A	d(D–H)	d(H···A)	d(D···A)	∠DHA	Symmetry codes
C(8)–H(8)···O(2)	0.93	2.27	3.115(12)	150	x,-1+y,z
C(23)–H(23)···O(4)	0.93	2.33	3.056(14)	135	x,1+y,z
C(28)–H(28A)···O(1)	0.97	2.40	2.830(13)	106	
C(29)–H(29A)···I(6)	0.97	3.03	3.823(9)	139	-1+x,1+y,z
C(14)–H(14B)···I(3)	0.97	2.86	3.772(11)	156	1-x,-1-y,1-z
